# Anatomic outcomes of laser indirect ophthalmoscopy for retinopathy of prematurity in a tertiary referral center in the Philippines

**DOI:** 10.1186/s13104-019-4303-3

**Published:** 2019-05-10

**Authors:** David Francis Fullon Chan, Milagros M. Herrera-Arroyo

**Affiliations:** Department of Ophthalmology and Visual Sciences, University of the Philippines Manila-Philippine General Hospital, Manila, Philippines

**Keywords:** Retinopathy of prematurity, ROP, Laser, Bevacizumab, Type 1 ROP, Philippines, Lower middle income, Developing country

## Abstract

**Objective:**

Laser/photocoagulation criteria for retinopathy of prematurity (ROP) are generally universal, with > 90% anatomic success reported in varied settings. Outcomes in the Philippines, a developing/lower-middle income nation, were examined.

**Results:**

This single-center retrospective chart review covered years 2014 and 2015. Of 214 infants screened, 64 had any ROP. Thirty-four were treated, and 20 had documented outcomes. Only 15 of 25 eyes (8 infants) with laser treatment-requiring ROP were successes (60%). All infants had Type 1 ROP except one with Aggressive Posterior ROP. Seven infants with bilateral “Milder than Type 1” ROP were treated successfully. Type 1 ROP treatment failures versus successes did not differ significantly in birth weight (1009 g vs. 1112.86 g, p = .5152), birth age of gestation (27.74 vs. 28.49 weeks, p = .3290), and, delay in first screening (6.74 vs. 5 weeks, p = .4649). Poorer outcomes of laser treatment-requiring ROP were documented here compared with elsewhere in the world. Variables/risk factors examined were limited and human error was not systematically considered. Subsequent studies validating this trend should incorporate other clinical (e.g. maternal/neonatal risk factors) and environmental differences that might drive pathology and treatment response in this multifactorial disease. Adherence to protocols gains importance given the widespread delay and early loss to follow-up observed.

**Electronic supplementary material:**

The online version of this article (10.1186/s13104-019-4303-3) contains supplementary material, which is available to authorized users.

## Introduction

Subject to multiple risk factors, retinopathy of prematurity (ROP) demands continuous re-examination of management paradigms. While majority of cases regress, differences between populations appear to drive pathology. For example, screening criteria constantly undergo reexamination, as data from less developed countries/populations suggest progression risk persists among their infants born more mature and heavier [1–3].

A developing (lower-middle income) country with steady economic expansion but persistently poor maternal and neonatal health outcomes relative to its growth, the Philippines was in a 2012 list of populations having high risk for blindness due to ROP because of inadequate neonatal care and screening [4–7]. Incidence rates in local hospitals have been reported at 13.8–25.9% [6, 8, 9]. Data from neighbors with varying ethnic, cultural and economic overlaps with the Philippines—Indonesia, Taiwan, Thailand—report up to 29.7–31.7% incidence [10–12]. None report treatment outcomes. All have screening criteria more inclusive of infants born more mature and heavier versus more advanced nations.

Criteria for laser photocoagulation (peripheral retinal ablation via laser indirect ophthalmoscopy, “LIO”) meanwhile is relatively stable and universal worldwide, determined by a diagnosis of Type 1 ROP per the Early Treatment for Retinopathy of Prematurity Cooperative Group (ETROP) study [13]. Success rates, even in less developed nations, exceed 90% [3, 14, 15]. Anti-vascular endothelial growth factor (VEGF) therapy has arisen, with the discovery that VEGF levels rise in phase 2 ROP. Protocols are evolving, as particulars of safety, timing, dosage, stability of effect are under study. Although success with monotherapy is being reported, local guidelines advise use for very aggressive ROP, whether as adjunct to LIO or primary treatment, with caution [2, 16, 17].

To the best of our knowledge, ROP treatment outcomes in the Philippines have not been reported. We thus examined the applicability of the said treatment criteria to our setting. Investigating if treatment response is consistent with that elsewhere, we examined anatomic outcomes following treatments on infants screened in a tertiary referral center over 2014 and 2015.

## Main text

### Methods

This was a single-center retrospective chart review conducted in the national university hospital, a tertiary referral center. Two hundred fourteen records of ROP patients referred to the Vitreoretina Service from January 1, 2014 to December 31, 2015 were secured.

#### Screening and treatment protocol

Screening (dilated indirect ophthalmoscopy) and treatment schedules followed local guidelines: screening is recommended for infants < 35 weeks birth age of gestation (AOG), or, having < 2000 g birth weight (BW), or, heavier and/or older but with stormy neonatal course [18]. First examination is recommended at 2 weeks post-natal age (PNA) or, 32 weeks postconceptional age (PCA = AOG + PNA), whichever comes earlier. LIO is indicated for Type 1 ROP and Aggressive Posterior ROP (AP-ROP). Intravitreal anti-VEGF injection is either adjunct to LIO, or, primary treatment with caution for aggressive ROP such as Zone 1 Stage 3+, or, AP-ROP.

Screening was conducted by medical retina fellows. Diagnoses followed International Classification of ROP (ICROP) standards [18]. No imaging was conducted. Documentation was by manual fundus drawing, and recording of diagnosis per ICROP. Confirmation of both diagnosis and treatment adequacy were done by consultant faculty. Repeat screening after 2–3 weeks was advised for cases of immature retina, and, after 2–7 days for ROP.

#### Treatment methodology

Treatment followed ETROP study specifications: near-confluent gray-white ablation of peripheral avascular retina using LIO [13]. Intravitreal Bevacizumab injection (IVB) followed BEAT-ROP methodology: .625 mg Bevacizumab in .025 ml of solution (AVASTIN, 100 mg/4 ml, Roche, Switzerland) [16]. Treated infants were examined daily, with interval prolongation only once with regression.

#### Anatomic outcomes

Unfavorable anatomic outcome/treatment failure was judged given fundus changes as described in ETROP-posterior retinal fold involving the macula, retinal detachment (Stage 4 or 5 ROP), or, retrolental tissue or “mass” obstructing view of the posterior pole [13]. Favorable anatomic outcome/treatment success was judged given ROP regression.

#### Data analysis

Baseline characteristics were reported using descriptive statistics. Complete case analysis was used for handling missing data. Mean BW’s and birth AOG’s of the normal (no ROP) and ROP groups were compared using T-test. Mann–Whitney U and Kruskal–Wallis tests were used to compare the same variables between the treatment successes, failures, and disease milder than Type 1 ROP groups. Stata 14.0 (College Station, TX) was used.

### Results

In the period 2014–2015, 214 infants were referred for ROP screening. Two of the infants with no ROP and 4 of the infants with ROP had either no BW or birth AOG, excluding them from further analysis. Infants with any ROP were born with lower birth AOG and BW compared to those without ROP. (29.50 vs. 32.74 weeks, 1178.33 g vs. 1618.79 g, and, both p ≤ .001) (Table 1, Additional file 1: Table S1).

**Table 1:** Clinical characteristics of all referred patients. (N = 214)

	Screening terminated, no ROP = 91	With any ROP = 64	No ROP as of last screening but lost to follow-up = 59
Sex, n			
Male	36 (39.56%)	35 (54.69%)	26 (44.07%)
Female	38 (41.76%)	23 (35.94%)	29 (49.15%)
Data not available	17 (18.69%)	7 (10.94%)	4 (6.78%)
Age of gestation, weeks	32.74^a^	29.50^b^	31.75^a^
Birth weight, grams	1618.78^c^	1178.33^d^	1389.51^d^
Delay in screening vs. guidelines, weeks	5.27^e^	6.03^f^	2.52 ^g^

p ≤ .001 comparing age of gestation and birth weight between No ROP and With Any ROP, t-test

^a^By pediatric aging/Ballard score

^b^By last menstrual period in nine cases, by Pediatric Aging/Ballard Score otherwise

^c^Not recorded in 2 infants

^d^Not recorded in 4 infants; BW’s were available for all treatment successes and failures

^e^Excludes 8 infants seen on time

^f^Excludes 2 infants seen on time, and, 1 infant seen 3 years after

^g^Excludes 5 infants seen on time

Of the 64 infants diagnosed with ROP, 34 were treated. All received laser, 5 received IVB. Ultimately however, only 20 infants returned for complete monitoring of treatment outcome, including 3 of the 5 IVB-treated infants. Treatment success was seen in 15 of 25 eyes (8 patients) or 60% with treatment-requiring ROP; all infants had Type 1 ROP except 1 with bilateral AP-ROP. One infant (Patient 1, Summary Table 3) had successful Type 1 ROP LIO in one eye, and, Stage 4A ROP in the other eye on first examination (Table 2, Additional file 1: Tables S2, S3).

**Table 2:** Treatment outcomes

	Patients	Eyes	Regressed/treatment success, eyes	Unfavorable/treatment failure, eyes
Type 1 ROP excluding AP-ROP	12	23	13	10
AP-ROP	1	2	2	0
Disease milder than Type 1 ROP	7	14	14	0

n = 20

Outcomes for IVB treatment were available for three cases of bilateral Type 1 ROP (Additional file 1: Table S4). At birth they were aged 26–29 weeks, weighing 900–1200 g. Treatment was at week 35–39 4/7 AOG. All had Stage 3 ROP with Plus, Zone 2, in both infants born 900 g, and Zone 1 in the infant born 1200 g. LIO was done 1–22 days after IVB. Treatment success was observed in the infant with median birth AOG, BW, and screening delay. This infant had satisfactory response to a single injection until laser was deemed necessary 22 days later. Both treatment failures were cases of IVB as adjunct to LIO.

In infants with ROP but without treatment, spontaneous regression was seen in only nine cases. Their average BW was 1324.38 g (unavailable for one infant) and AOG 31.33 weeks.

### Discussion

Our laser treatment-requiring ROP success rate of 60% (15 of 25 eyes) contradicts the trend of good outcomes (> 90%) elsewhere—inferior even to CRYO-ROP study infants (68.9% success using cryotherapy) where the older “threshold” criteria were applied [2, 3, 13–15, 19]. Bearing in mind limitations mentioned later, this may be the first documentation of poor outcomes for this treatment paradigm/modality.

Treatment success and failures did not differ significantly along variables documented (Table 3). Failures had lower average BW’s (1009 g vs. 1112.86 g, p = .5152). Despite a lack of uniformity in reporting birth AOG (11 infants were aged by Ballard score, which can estimate AOG ±2 weeks of true AOG), failures also tended to be born more premature than successes (27.74 vs. 28.49 weeks, p = .3290) [20, 21]. They were also first screened with greater delay (6.74 vs. 5 weeks, p = .4649).

**Table 3:** Mean birth weights and birth AOG of treated patients

	Mean birth weight (g)	Mean birth AOG (weeks)
Type 1 ROP excluding AP-ROP with treatment outcomes n = 12 patients, 23 eyes	1069.58 (715–1490)	28.18 (26–31)
Treatment successes n = 7 patients, 13 eyes	1112.86 (900–1490)	28.49 (27–31)
Treatment failures n = 5 patients, 10 eyes	1009 (715–1300)	27.74 (26–30 4/7)
p, treatment successes vs. failures, Mann–Whitney U	.5152	.3290
Disease milder than Type 1 n = 7 patients, 14 eyes	1344.29 (850–2000)	29.82 (27-34)
p, comparison of median values, all three groups. Kruskal–Wallis	.3492	.2856

A multifactorial disease, subtle differences in AOG and BW probably combine with other characteristics imparting greater odds for poorer outcomes. The first is race, as associations between Asian race and predisposition to more severe disease are being discovered [11]. Next are poorer baseline health and socioeconomic status. Ours is the largest tertiary referral center in the country; a public institution where most patients fit into this profile. While this may constitute selection bias favoring poorer outcomes, it allows greater approximation of the profile of interest. Crucially, poor record-keeping prevented documentation and analysis of pre- and perinatal infant and maternal risk factors.

Finally, human error must be considered. Although confirmation of diagnosis by consultant faculty occurred no later than 2 days after initial diagnosis, and, average diagnosis-to-treatment time was 2.5 days (0–7 days), cumulative delay may have resulted. Although treatment was on time per guidelines, it may have occurred later into the disease process. Additional laser sittings were done in two each of the treatment successes and failures. Inadequate treatment in any of the failures cannot be absolutely ruled out.

Fellows change yearly, and their learning curve might result in delays and inadequate treatment. Errors in consultant diagnosis and assessment of treatment adequacy are also possible. In this cohort, verification is not possible with the absence of objective records (imaging). Greater supervision and validation of fellows’ competencies through objective tests and certifications, and, access to objective documentation systems may be of value for research and improving outcomes. Should competence be established, unique treatment response in this population can be considered more.

Ultimately however, assignment of root cause(s) requires a study design sufficiently covering all the aforementioned. For now however, these findings prompt concern over the adequacy of protocols and possibly also those in similar populations. Firstly, the degree of late screening and poor/loss to follow-up is unacceptable. Only 15 infants underwent first examination/screening on time. The rest were delayed by 4.86 weeks on average beyond mandated first screening date. Incomplete screening occurred for 89 infants (59 without ROP as of last screening, 16 with ROP lost untreated and without outcome documented, 14 treated but lost before outcome documented). With such data loss, it is possible our 29.91% incidence rate of any ROP—roughly similar to neighboring nations mentioned—is higher, and, our treatment outcomes poorer. Most tellingly, 5 infants had some traction or detachment on first screening. Measures to increase awareness of and adherence to screening recommendations appear necessary.

Lowering treatment thresholds is an attractive stop-gap measure, particularly with the treatment success seen in all seven patients who were treated for disease milder than Type 1 ROP (Additional file 1: Table S5). They demonstrated features not recognized by guidelines, sometimes akin to the entity of “smouldering ROP:” tortuous and dilated terminal vessels (terminal Plus), loss of the dichotomous branching of retinal vessels, gray/ischemic-looking anterior retina, and, peripheral vessel circumferential closure [18, 22, 23]. Treatment was favored due to the atypical nature of findings, and estimated poor ability to return for follow-up. Compared to treatment successes and failures, their average BW was higher (1344.29 g), and birth AOG later (29.82 weeks). Median BW’s and AOG’s of all groups were not significantly different from each other (p = .3492 and .2856 respectively) however (Table 3).

All cases were milder even than Type 2 ROP—a group in ETROP with only 2.1% unfavorable anatomy at 6 months [13]. As suggested by a multicenter review, this may demonstrate how individual judgment has a role in unique situations [23]. However, loss of peripheral vision and induction of error of refraction are well-established LIO side effects [24]. Adjusting officially recommended treatment thresholds towards this paradigm should perhaps await a more complete picture of local outcomes.

### Conclusion

Data from this 2-year review found evidence of Type 1 ROP laser treatment success rates worse than in multiple populations [2, 3, 13–15, 19]. While characteristics of the treatment failures were not significantly different from other groups, they were oriented towards lower BW, greater prematurity, and more delayed screening. Subsequent studies are needed to validate these outcomes, and if consistent, identify variables that push eyes towards progressive, more treatment-resistant ROP. They may incorporate closer scrutiny of the variables examined, in light of limitations stated. As late screening and treatment are followed by high and early loss to follow-up in almost all patients, local incidence of ROP could be higher, and, outcomes worse than reported. Adherence to and re-examination of evidence-based guidelines must be encouraged, more urgently so in our and similar populations facing persistently challenging public health environments [7].

### Limitations

Gaps and insufficiencies in the data were critical limitations. Cohort and subgroups were small, and, pre- and perinatal risk factor data was scant, compromising strength of quantitative analyses, and the ability to investigate interactions of multiple factors, which is appropriate/necessary for this disease. Uniformity in format, as in age estimation, may have reduced accuracy of data. Diagnoses and assessments of treatment adequacy had no objective documentation such as imaging, and are thus subject to human error. This is in addition to the learning curve of fellows-in-training. Retrospective design and limitation to a single center constrain analyses, generalizability, and potential to question the efficacy of the treatment paradigm in general. Adherence to and refinement of protocols, and, subsequent validation of the findings appear necessary.

## Additional file

**Additional file 1.** The additional file contains all Additional Tables S1 to S5, of which the latter 4 are on landscape-oriented pages due to width. They contain datasets on individual patients that may be useful for further scrutiny both by reviewers and readers.

## Data Availability

The datasets used and/or analyzed during the current study are available from the corresponding author on reasonable request.

## Abbreviations

AOG: age of gestation
AP-ROP: Aggressive Posterior-ROP
ETROP: Early Treatment for Retinopathy of Prematurity Cooperative Group
IVB: intravitreal Bevacizumab injection
LIO: laser indirect ophthalmoscopy
PNA: post-natal age
ROP: retinopathy of prematurity
VEGF: vascular endothelial growth factor
