# Supplementary material for: Anatomic outcomes of laser indirect ophthalmoscopy for retinopathy of prematurity in a tertiary referral center in the Philippines
Source: BMC Res Notes. 2019 May 10;12:263. doi: 10.1186/s13104-019-4303-3 (PMC6511119; doi:10.1186/s13104-019-4303-3)
Supplement: Supplementary file 1 — Additional file 1. The additional file contains all Additional Tables S1 to S5, of which the latter 4 are on landscape-oriented pages due to width. They contain datasets on individual patients that may be useful for further scrutiny both by reviewers and readers. [file 13104_2019_4303_MOESM1_ESM.docx]

**Table S1***: Outcomes of patients with any ROP. (n=64)*

|  | **patients** |
| --- | --- |
| treated with laser or intravitreal Bevacizumab | **34** |
| *treated, outcome documented* | *20* |
| *treated, lost to follow-up before outcome documented* | *14* |
| spontaneous regression OU | **9** |
| lost to follow-up untreated and without outcome documented | **16** |
| unfavorable OU on first consult | **5** |

**Table S2***: Type 1 ROP treatment failures.*

| Patient number | Sex (0 unavailable, 1 male, 2 female) | Age of Gestation/AOG (weeks) | Birth Weight (grams) | ROP Location (Zone) | ROP Stage | Threshold | AOG at first treatment (weeks) | Outcome | Treatment, sequence (days after previous treatment) | Time from last treatment to documentation of failure (weeks) | AOG on documentation of failure (weeks) | delay of screening (weeks) | Maternal or neonatal risk factors |
| --- | --- | --- | --- | --- | --- | --- | --- | --- | --- | --- | --- | --- | --- |
| 8 | 1 | 29 | 1200 | OU: I | OU:3 | OU: + | 35 | OU: failure | 1. IVB  2. laser (7) 3. laser (4) | 4 1/7 | 40 4/7 | 2 | 0 |
| 9 | 2 | 26 | 900 | OU: II | OU:3 | OU: + | 39 4/7 | OU: failure | 1. IVB  2. laser (1) 3. laser (1) | 3/7 | 39 6/7 | 11 2/7 | 0 |
| 10 | 0 | 26 1/7 | 715 | OU: I | OU:3 | OU: + | 37 2/7 | OU: failure | Laser | 4/7 | 37 6/7 | 9 | 4,5,7,8 |
| 11 | 1 | 30 4/7 | 930 | OU: I | OU:3 | OU: + | 37 2/7 | OU: failure | Laser | 2 2/7 | 39 4/7 | 5 3/7 | 3,5,10 |
| 12 | 2 | 27 | 1300 | OU: II | OU:3 | OU: + | 36 | OU: failure | Laser | 1 | 37 | 6 | 0 |

*Maternal or neonatal risk factors:*

*0 no records available*

*1 maternal urinary tract infection*

*2 maternal hypertension*

*3 maternal heart disease*

*4 premature rupture of membranes*

*5 nasal continuous positive airway pressure*

*6 ventilator use/endotracheal intubation*

*7 pneumonia*

*8 sepsis*

*9 necrotizing enterocolitis*

*10 blood transfusion*

| Patient number | Sex (0 unavailable, 1 male, 2 female) | Age of Gestation/AOG (weeks) | Birth Weight (grams) | ROP Location (Zone) | ROP Stage | Threshold | AOG at treatment (weeks) | Outcome | Treatment, sequence (days after previous treatment) | Follow-Up after last treatment (weeks) | AOG as of last funduscopy (weeks) | delay of screening (weeks) | Maternal or neonatal risk factors |
| --- | --- | --- | --- | --- | --- | --- | --- | --- | --- | --- | --- | --- | --- |
| 1 | 2 | 28 3/7 | 1105 | OD: II | OD: II | OU + | 38 3/7 | OD: success | 1. laser  2. laser (5) | 10 5/7 | 51 6/7 | 8 1/7 | 0 |
| 2 | 0 | 27 | 900 | OU: II | OU:3 | OU + | 39 2/7 | OU: success | 1. IVB 2. laser (22)  3. laser (8) | 26 4/7 | 68 | 9 5/7 | 0 |
| 3 | 2 | 27 2/7 | 1000 | OU: II | OU:2 | OD + OS - | 38 | OU: success | Laser | 24 | 63 1/7 | 6 6/7 | 1,6,7,8 |
| 4 | 1 | 29 6/7 | 1150 | OU: I | OU:2 | OU + | 38 6/7 | OU: success | Laser | 44 3/7 | 83 2/7 | 4 2/7 | 2,5,8,9,10 |
| 5 | 1 | 28 | 1115 | OU: I | OU:3 | OU + | 34 3/7 | OU: success | Laser | 23 | 57 6/7 | 3 6/7 | 0 |
| 6 | 1 | 31 | 1490 | OU: II | OU:3 | OU + | 35 5/7 | OU: success | Laser | 7 2/7 | 43 2/7 | 2 4/7 | 0 |
| 7 | 0 | 27 6/7 | 1030 | OU: II | OU:3 | OU + | 32 2/7 | OU: success | Laser | 17 2/7 | 48 4/7 | 0 | 1,5 |
| 20 | 0 | 30 | 930 | (AP-ROP) | (AP-ROP) | (AP-ROP) | 36 5/7 | OU: success | Laser | 14 2/7 | 51 | 4 | 0 |

**Table S3***: Type 1 ROP treatment successes.*

*Maternal or neonatal risk factors:*

*0 no records available*

*1 maternal urinary tract infection*

*2 maternal hypertension*

*3 maternal heart disease*

*4 premature rupture of membranes*

*5 nasal continuous positive airway pressure*

*6 ventilator use/endotracheal intubation*

*7 pneumonia*

*8 sepsis*

*9 necrotizing enterocolitis*

*10 blood transfusion*

| Patient | Sex (0 unavailable, 1 male, 2 female) | Age of Gestation/AOG (weeks) | Birth Weight (grams) | ROP Location (Zone) | ROP Stage | Plus | AOG at first treatment (weeks) | Latest assessment | Threshold | Treatment, sequence (days after previous treatment) | Time from last treatment to latest assessment (weeks) | AOG at latest assessment (weeks) | delay of screening (weeks) |
| --- | --- | --- | --- | --- | --- | --- | --- | --- | --- | --- | --- | --- | --- |
| 2 | 0 | 27 | 900 | OU: II | OU:3 | + | 39 2/7 | OU: success | OU + | 1. IVB 2. laser (22)  3. laser (8) | 26 4/7 | 68 | 9 5/7 |
| 8 | 1 | 29 | 1200 | OU: I | OU:3 | + | 35 | OU: failure | OU + | 1. IVB 2. laser (7) 3. laser (4) | 4 1/7 | 40 4/7 | 2 |
| 9 | 2 | 26 | 900 | OU: II | OU:3 | + | 39 4/7 | OU: failure | OU + | 1. IVB  2. laser (1) 3. laser (1) | 3/7 | 40 3/7 | 11 2/7 |

**Table S4***: Intravitreal Bevacizumab (IVB) treated patients.*

*(Note: No maternal or neonatal risk factor data/records were available for all these infants.)*

| Patient | Sex (0 unavailable, 1 male, 2 female) | Age of Gestation/AOG (weeks) | Birth Weight (grams) | ROP Location (Zone) | ROP Stage | Plus | AOG at first treatment (weeks) | Outcome | Treatment, sequence (days after previous treatment) | Follow-Up after last treatment (weeks) | AOG as of last funduscopy (weeks) | delay of screening (weeks) | Latest assessment | Indication for treatment |
| --- | --- | --- | --- | --- | --- | --- | --- | --- | --- | --- | --- | --- | --- | --- |
| *13* | 1 | *27* | *1010* | *OU: II* | *OU:2* | *-* | 41 2/7 | *OU: success* | *laser* | 2 3/7 | 45 5/7 | *1 2/7* | *success* | gray and ischemic-looking anterior retina, marked multiple branching at vessel termini |
| *14* | 1 | *27 2/7* | *1390* | *OU: III* | *OU:2* | *-* | 40 1/7 | *OU: success* | *laser* | 1 | 42 1/7 | *1 1/7* | *success* | tortuous, dilated terminal vessels |
| *15* | 1 | *32* | *1360* | *OU: II* | *OU:1* | *-* | 53 1/7 | *OU: success* | *laser* | 6 1/7 | 59 2/7 | *19* | *success* | tortuous, dilated terminal vessels |
| *16* | 1 | *32* | *1800* | *OU: II* | *OU:3* | *-* | 40 2/7 | *OU: success* | *laser* | 8 6/7 | 49 1/7 | *6 6/7* | *success* | tortuous, dilated terminal vessels |
| *17* | 2 | *28 3/7* | *850* | *OU: III* | *OU:3* | *+* | 38 1/7 | *OU: success* | *laser* | 92 3/7 | 130 4/7 | *7 4/7* | *success* | tortuous, dilated terminal vessels |
| *18* | 2 | *28* | *1000* | *OU: III* | *OU:3* | *-* | 41 2/7 | *OU: success* | *laser* | 9 4/7 | 50 6/7 | *11 1/7* | *success* | persistent Stage 3 ROP despite advanced age |
| *19* | 1 | *34* | *2000* | *OU: III* | *OU:2* | *-* | 51 6/7 | *OU: success* | *laser* | 9 5/7 | 61 2/3 | *2 5/7* | *success* | circumferential closure, persistent Stage 2 ROP despite advanced age |

**Table S5***: Mean birth weights and birth AOG of treated patients with Disease Milder than Type 1 ROP.*

*Maternal or neonatal risk factor were present only for patient 17 (nasal continuous positive airway pressure and pneumonia). No records could be found for all other infants*
